# Scientometric analysis of the world-wide research efforts concerning Leishmaniasis

**DOI:** 10.1186/1756-3305-3-14

**Published:** 2010-03-04

**Authors:** K Al-Mutawakel, C Scutaru, A Shami, M Sakr, DA Groneberg, D Quarcoo

**Affiliations:** 1 Department of Dermatology and Venerology, Sana'a University Faculty of Medicine and Dentistry, Sana'a, Yemen; 2 Institute of Occupational Medicine, Charité-Universitätsmedizin Berlin, Free University Berlin and Humboldt-University Berlin, Germany

## Abstract

**Background:**

Leishmaniasis is a chronic disease that is found in various countries of the world. The aim of the current study was to investigate the impact of leishmaniasis on the world's research output. The present study assessed benchmarking of research output for the period between 1957 and 2006. Using large database analyses, research in the field of leishmaniasis was evaluated. Furthermore, cooperation between different countries was identified.

**Results:**

The number of publications increased with time. Most publications came from Western countries such as the US, UK or Germany. Interestingly, countries like Brazil and India had a high research output. We found a substantial amount of cooperation between countries.

**Conclusion:**

Although leishmaniasis is of limited geographic distribution it attracts a wide research interest. The central hub of research cooperation is the USA.

## Background

Good willed policy-makers in the world have the eminent goal to improve the life of their people. In the medical sector, scientific research leading to new prevention and treatment strategies has the potential of enormous social value for the population [1]. A widely used instrument to enhance research output is to allocate funds to the institutions that provide scientific progress [2,3]. Because funding resources are limited in most countries, decision-makers have to select areas and projects that are worthy of support, taking into account, besides other factors, the urgency of the anticipated results. The decision to support a research project might depend on other reasons such as the prevalence of a disease. Also, the acute and chronic severity of that disease might influence the motivation to embark on research projects in this field. In recent years, tools have been developed that assist in finding parameters that can be used to prioritise a list of proposals, embracing the particular characteristics of a research field in retrospective. One such tool is the bibliometric analysis of medical subjects [4]. By objectively describing the research activities of a given country, it provides an evaluation of the strength and weakness throughout a time period. To examine how different influences shape the scientific output in different countries we choose leishmaniasis, a disease that is found in tropical, subtropical or temperate zones of more than 80 countries [5]. Each year there are reports of 1-1.5 million new cases worldwide [6]. India has the highest burden of disease worldwide. In South America, Brazil reports the highest number of infections with leishmaniasis [7]. The disease is caused by a protozoan, of the genus *Leishmania *and is transmitted by the bite of some species of sand flies [8]. The name of the disease was given after the Scottish physician William Boog Leishman, who independently from Charles Donavan found characteristic bodies in the spleen of patients who suffered from the disease [9]. Leishmaniasis has several names depending on geography such as leishmaniosis, leishmaniose, Oriental Boils, Baghdad Boil, kala azar, black fever, sand fly disease, Dum-Dum fever or espundia [10]. From 30 species which infect mammals, 21 have the potential to infect humans. The most severe form of leishmaniasis is visceral leishmaniasis in which the parasites migrate to the vital organs [11].

Although, there has been a global interest in leishmaniasis this is the first study that analyses the global impact of this disease on the world's biomedical research.

## Methods

### Data source

The database used for the analysis was the Web of Science database from the Thomson Institute for Scientific Information (ISI) [12,13].

### Search strategies

For the searches the term "leishman*" was used. The "*" is a wildcard which can take any value. This is needed for finding all the articles which are related to leishmaniasis. The present study was designed to assess the overall number of publications related to leishmaniasis.

### Time span

The initially analyzed time span included the period from 1957 to 2007. To some extent the time span was limited to the years which contained at least 30 articles, in order to be able to perform statistics like the average citation per item.

### Software analysis

The query into the ISI database returned all the articles which matched our criteria. These articles were downloaded using the download function of the web interface as "Full Article" and in "Plain Text" format. A C++ software parsed the text files and inserted the information into a relational database for further analysis. The following information was retrieved from the text files:

• Publication year,

• Total times the article has been cited

• Country of origin of the different authors

• Number of authors

### Data analysis

Using the "Analyze" function provided by the ISI database the article were sorted according to the language in which the article has been written.

The C++ software computed following information from the database:

• Year analysis: total number of published items, total number of citations, average number of citations per item, distribution of cooperation articles over the time span, evolution of the mean number of authors per article over the time span

• Country analysis: total number of published items, total number of citations, average number of citations per item, cooperation level between the countries.

The cooperation between two countries A and B was defined as the total number of articles where at least one author originates from A and at least one from B. The order of the authors as well as the presence of other countries in the articles has no impact on this figure. Each article is counted in accordance to the number of countries that worked on it.

### Density-equalizing mapping

Density equalizing mapping was used according to a recently published method and as previously described [14]. In brief, the territories of the contributing countries were re-sized according to a particular variable, i.e. the number of published items or the average citation per published item; in the final projection of the map the countries have the same density per square kilometer of the given variable. The specific calculations are based on Gastner and Newman's algorithm [15]. The following cartograms were generated from the gathered data:

• Distribution of the articles over the world

• Average citation per item (only computed for the countries with at least 30 published items)

## Results

### Number and citation of articles over time

**Figure 1:**
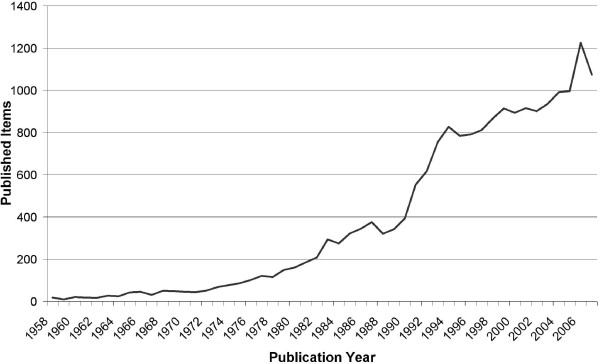
**Published items related to Leishmaniasis: total number in the Web of Science database between 1957-2007**.

The query returned 19277 articles which were downloaded from the ISI database. Figure 1 shows the distribution of the articles over the time span. There is a continuous increase in the number of articles per year with the most productive years being 2006 with 1226 published items, followed by 2007 (1075) and 2005 (996).

Looking at the average citation per item, the year 1993 has yielded the highest average citation per item with a value of 41.73, followed by 1992 with 37.47 and 1994 with 32.32. After the year 1993 the curve has a steep decline, however the articles published in these years will be most probably cited in the future. Only years with at least 30 articles were analyzed in order to filter out mavericks.

### Number of authors

**Figure 2:**
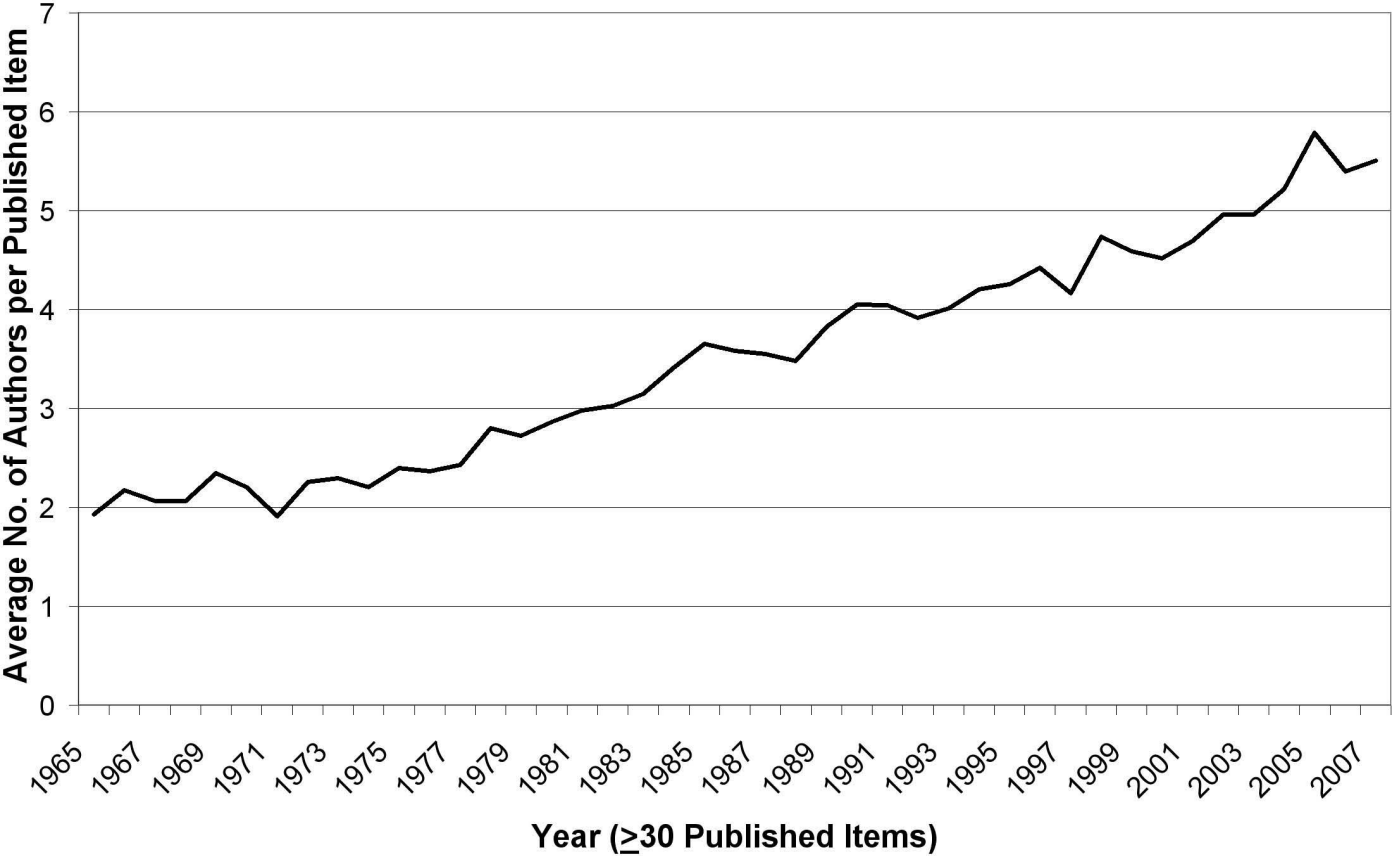
**Evolution of average number of authors per article**. The average number of authors that contributed to a published item is determined for each year.

The average number of authors per published item is presented in the Figure 2. Only years with at least 30 articles were analyzed. A continuous increase in the number of authors, starting in 1965 (with a value of 1.93 authors per publication and ending in 2007 with 5.51 authors per publication) which also represents the maximum value of the entire time span, can be seen.

### Distribution of countries

**Figure 3:**
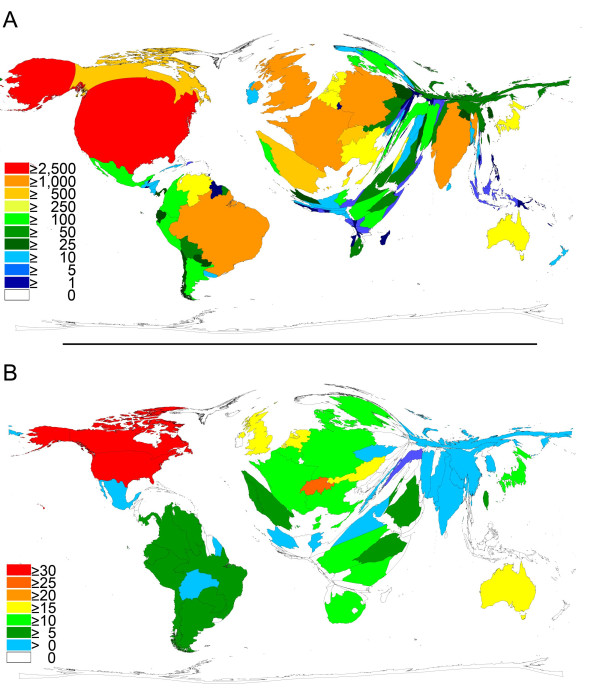
**Density-equalizing calculations**. A: Map illustrating the number of Leishmaniasis publications in each particular country. The area of each country was scaled in proportion to its total number of leishmaniasis publications. Color-coding visualizes total number of publications. B: Map showing the average citations per item of each country. The area of each country was scaled in proportion to its average number of citations per item. Color coding visualizes average citations per published item.

**Figure 4:**
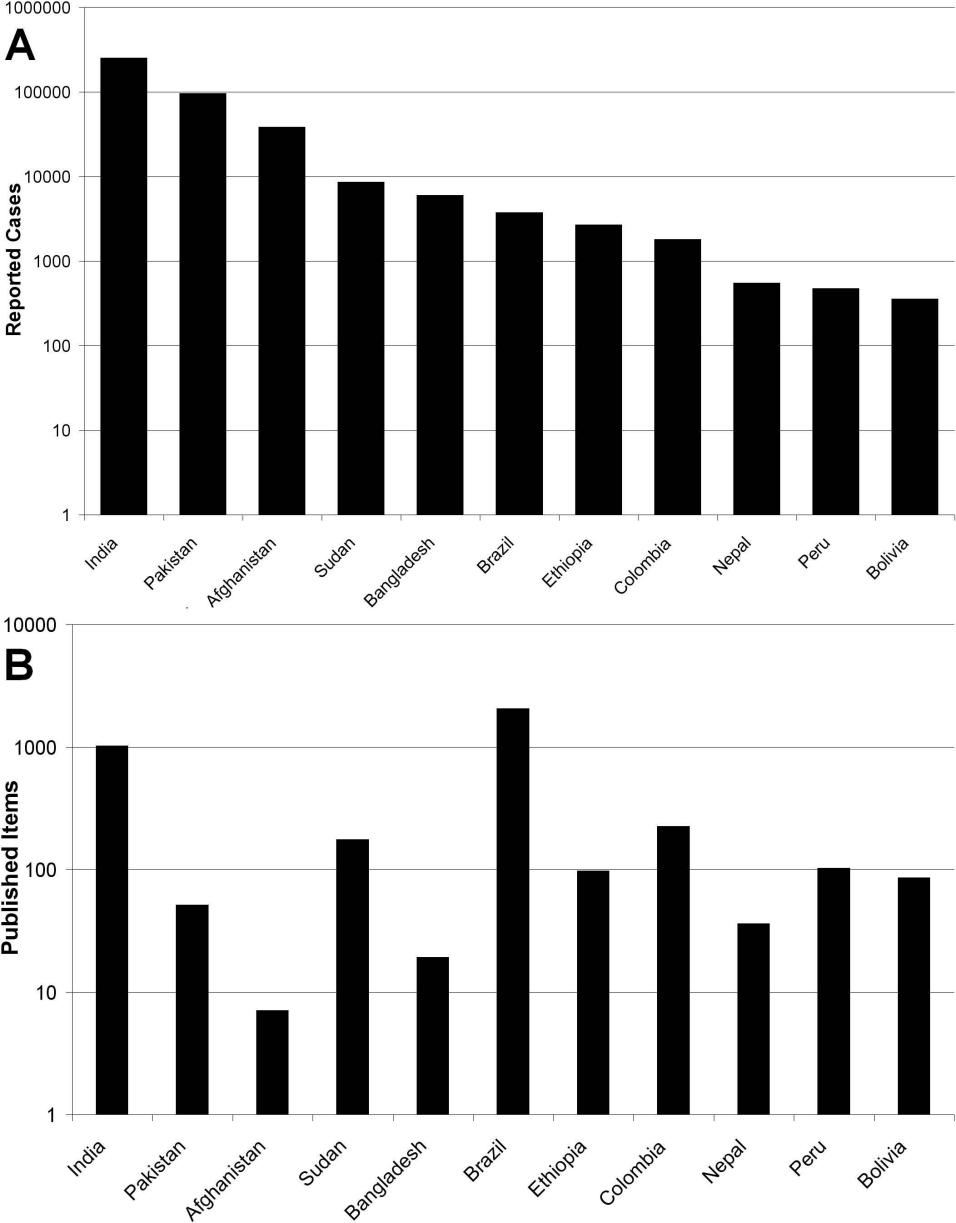
**Distribution of articles in relation to number of cooperating countries**. Of all cooperating articles the amount of different countries (2-13) involved in the research is depicted.

The 19277 articles originated from 140 different countries which makes an average of 137.7 articles per country. The top 10 productive countries are the United States of America (4905) followed by Brazil (2049), United Kingdom (1986), France (1281), India (1011), Germany (1002), Spain (833), Canada (597), Switzerland (545) and Italy (466). Figure 3A depicts the density equalizing map of the world where the surface of the country is correlated to the number of published items. Most articles are concentrated in North and South America (Brazil) and Western Europe. In Asia, India clearly holds the first place. Figure 4 puts side by side the incidence of leishmaniasis in countries with high disease burden with the amount of publication in these countries.

### Citing of articles

Looking from the perspective of the total citations, the top 10 countries slightly change the order as follows: United States of America (160802), United Kingdom (47533), Brazil (25876), France (21301), Germany (19619), Canada (18496), Switzerland (14069), Spain (10219), India (9910) and Italy (7214) (see Table 1). Using the average citation per item, the quality of the articles coming from a given country was assessed. Only countries with at least 30 articles were analyzed. This restriction leaves only 48 countries for analysis. The top 10 are United States (32.78), Canada (30.98), Switzerland (25.81), United Kingdom (23.93), Australia (22.94), Netherlands (22.28), Austria (20.18), Japan (19.71), Sudan (19.64) and Germany (19.58). Figure 3B shows the density equalizing map according to these figures. It is interesting to find an African country (Sudan) among the top 10.

**Table 1:** Total citations

Country	Total citations
United States	160802

United Kingdom	47533

Brazil	25876

France	21301

Germany	19619

Canada	18496

Switzerland	14069

Spain	10219

India	9910

Italy	7214

Australia	6812

Japan	6742

Netherlands	6705

Israel	6148

Venezuela	5037

Belgium	4895

Kenya	3417

Sudan	3417

Sweden	2794

Denmark	2657

Colombia	2424

Argentina	1954

Iran	1370

Portugal	1356

Mexico	1239

### Cooperation between countries

**Figure 5:**
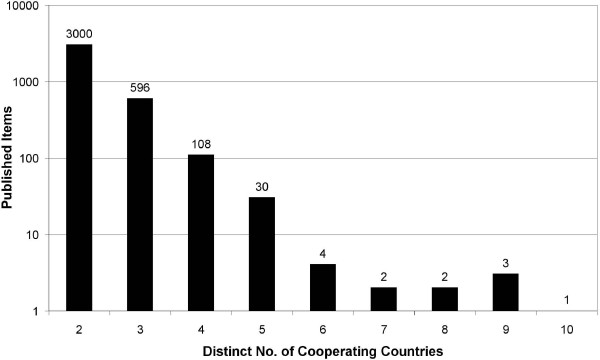
**Amount of cooperating articles**. The amount of articles that were produced by two or more countries is shown over time (1990 - 2007). A continuous increase can be noted.

**Figure 6:**
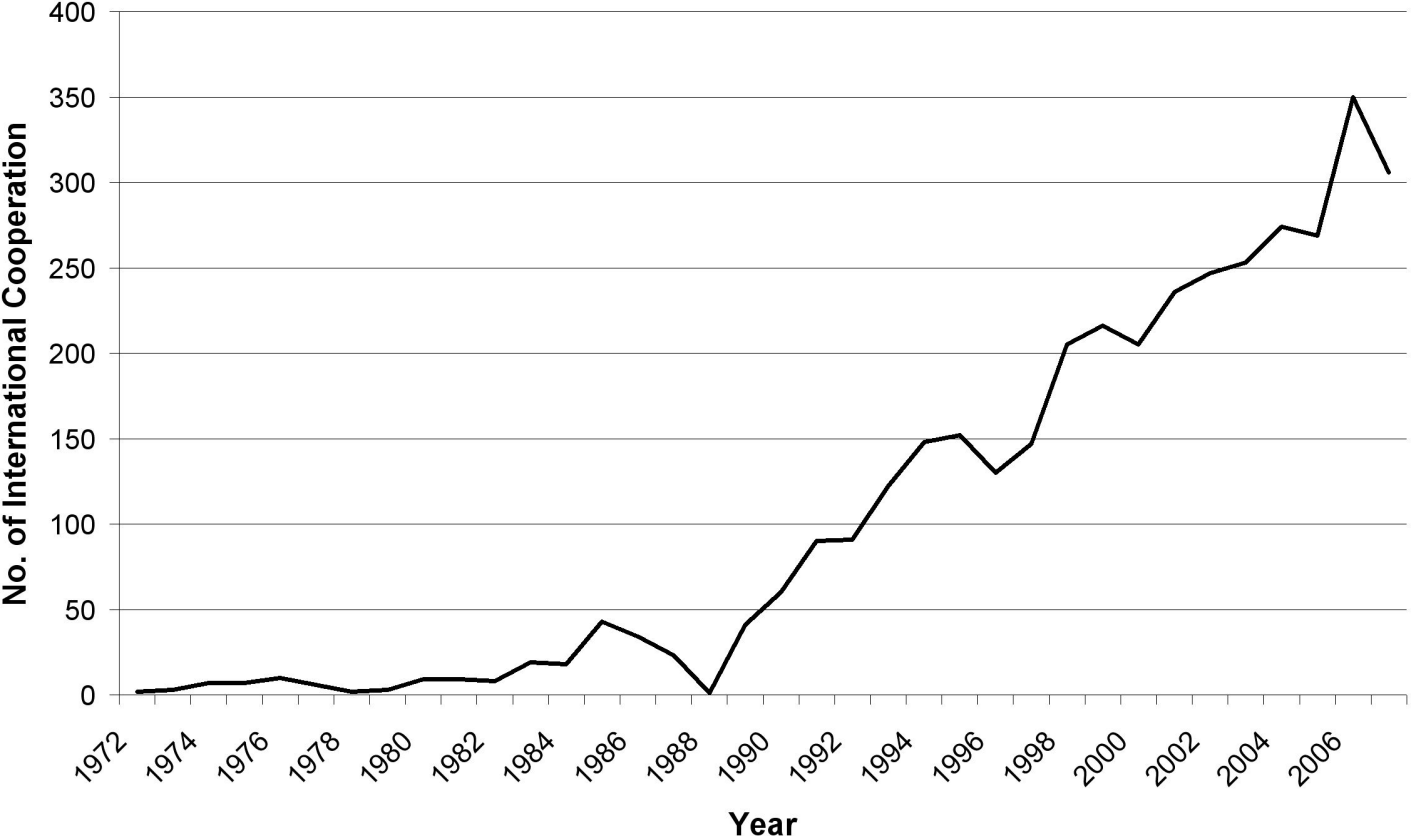
**Radar chart of cooperation density**. Grayscale encodes number of cooperation between countries.

**Figure 7:**
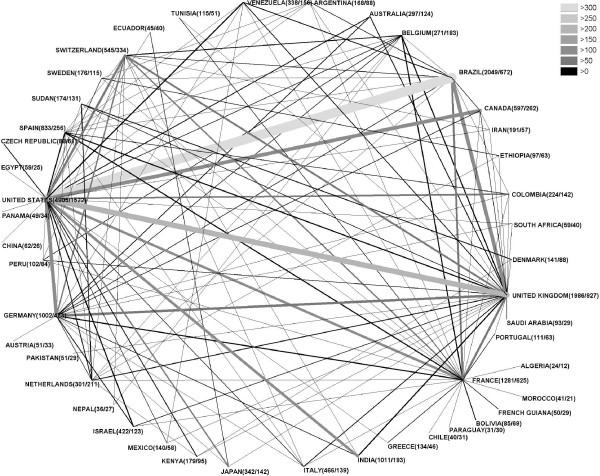
**Average number of authors per published item**. Average number of authors that worked on one article is illustrated over time (1957-2007). A continuous increase of authors over time can be noted.

Out of the 19277 articles only 3747 articles are the result of an international cooperation (19.43%). From all 140 countries 134 are present in cooperation. 80.09% of the cooperation articles (3000) are cooperation between only 2 countries, 18.91% (596) between 3 countries and 2.88% (108) are cooperation between 4 distinct countries (see Figure 5). Figure 6 depicts the evolution of the cooperation articles over the years. There is a steep increase starting with 1972 (2 articles) up to 306 in 2007 with a maximum in 2006 (350). In figure 7 the cooperation data are presented as radar chart. For readability reasons only cooperation's values of 30 and above are shown in the graph. Table 2 contains the top 25 cooperation. Looking at the first 10 cooperation values, the United States of America are present 7 times, the United Kingdom 5 times, Brazil and France 3 times, Germany and Switzerland 2 times and Canada and India 1 time. The highest level of cooperation is found between the United States of America and Brazil (364 articles) followed by United States and United Kingdom (215) and Canada with the United States (148) (see additional files 1, 2, 3 and 4).

**Table 2:** Cooperation

Pos	Country	Country	Cooperation
1	Brazil	United States	364

2	United Kingdom	United States	215

3	Canada	United States	148

4	Brazil	United Kingdom	145

5	Germany	United States	134

6	France	United States	103

6	India	United States	103

7	France	United Kingdom	92

8	Germany	United Kingdom	91

8	Switzerland	United States	91

9	Switzerland	United Kingdom	79

10	Brazil	France	71

11	Colombia	United States	63

12	Japan	United States	58

13	Germany	Switzerland	54

14	France	Switzerland	52

15	France	Spain	47

15	United States	Venezuela	47

16	Spain	United Kingdom	46

17	Israel	United States	44

18	India	United Kingdom	43

19	Spain	United States	42

20	Belgium	France	41

20	Belgium	United Kingdom	41

20	France	Germany	41

21	Bolivia	France	39

22	Australia	United States	38

22	Italy	United Kingdom	38

23	Brazil	Venezuela	37

23	Kenya	United States	37

23	United Kingdom	Venezuela	37

24	Australia	United Kingdom	35

24	Netherlands	United States	35

25	Denmark	Sudan	34

25	Netherlands	United Kingdom	34

## Discussion

The present study describes the first scientometric analysis of the parasitic disease of leishmaniasis. The disease has drawn global research interest documented by articles from 140 countries. Most articles came from countries with traditional high research output in North America and Europe, but also countries like Brazil and India have embarked on important research activity in this area. India is the country with the highest morbidity, so it is understandable to find it among the top publishing countries. Other countries with high morbidity cannot be found among the most publishing countries; this might be explained by the fact that these countries have a low income per capita (Figure 4) [16]. A good level of cooperation can be found between the countries, with the USA being a focal point of collaboration.

From the beginning of the investigated period, a steady increase of publication concerning leishmaniasis to over 1000/year was apparent. In 1963 de Sola Prince published a theory claiming that the amount of scientific output will double every 10-15 years [17]. In the field of leishmaniasis research we found an even steeper increase in publication, which might be due to the fact that in this small, isolated scientific area a self enhancing effect increases output further.

With the number of publications the number of authors/-publication also have increased over time. This phenomenon was also described by the above mentioned author [17] and has lead to articles with more than hundred co-authors in other research areas [18]. The worldwide increase in publication might partly be attributed to the advance in computer-based communication and association with the rapid development of the world-wide-net after 1990. This expansion also facilitated the cooperation between workgroups from different countries. Although various previous scientometric studies have documented a strong collaboration between the USA and the UK, in leishmaniasis research, the teamwork between USA and Brazil institutes is the strongest in the field [4].

The motivation for the great research involvement of the countries of North America and Europe does not inevitably follow similar lines, since for the population of these countries leishmaniasis is not a health problem. Next to altruistic reasons and the wish, in an ever coalescent world, to protect one's own population against introduced tropical diseases [19], the belief exists, that a pathological principle can be detected from dissecting the pathophysiology of the disease. In this line leishmaniasis has been used as disease model for the propensity of the parasite to survive the normal cellular clearing mechanism. Besides the countries with a traditional high level of research activities, countries with a high burden of disease put a great effort into this field [7]. Brazil ranked second in overall research output, whereas in a bibliometric study comparing the research activities of various organ systems (heart, lung liver etc.) covering 8 of 10 most prevalent chronic diseases worldwide, Brazil only ranked 19th. Also, India, in the current study ranks 9., clearly further back in the study of organ systems [14,20]. In these two countries research output is associated with incidence of the disease. Brazil leads together with the United States of America the cooperation in this research field.

The analysis of the entire available data on leishmaniasis is unique. However, it should be noted that every database research houses limitations. In the present case, we were not able to differentiate the publications according to different forms of the disease. It would have been interesting to see whether the Brazilian research output would have been influenced by the high incidence of cutaneous leishmaniasis in contrast to India with a higher incidence of the visceral form. Also, the issue of linguistic differences and its effects on publication quantity should be addressed. In this respect, the present study included the analysis of publications in all languages included in the data bases. The majority of publications are published in English and it is difficult for non-English journals to be included in the data bases. Therefore, numerous scientific publications in non-English languages are not accessible by the present approach. This is a major bias. English speaking countries such as the US, Canada or the UK have an advantage. However, it is generally accepted that scientists from non-English speaking countries in Europe and Asia publish their high quality research in scientific journals that use English as language.

In conclusion we have found that although leishmaniasis is of defined geographic range it draws a wide research interest. The central hub of research cooperation is the USA.

## Competing interests

The authors declare that they have no competing interests.

## Authors' contributions

A-MK participated in the design and co-ordination of the study, performed the analysis, and drafted and prepared the manuscript. SC, SM and SA and participated in the analysis, GDA helped to interpret the data. DQ conceived of the study and helped to interpret the data and to prepare the manuscript. All authors read and approved the final manuscript.

## Supplementary Material

Additional file 1 **Fig 8.1**. All cooperative articles that were produced between 1957 and 2007 are shown, (countries A-L/A-M). Click here for file

Additional file 2 **Fig 8.2**. All cooperative articles that were produced between 1957 and 2007 are shown, (countries A-L/M-Z). Click here for file

Additional file 3 **Fig 8.3**. All cooperative articles that were produced between 1957 and 2007 are shown, (countries M- Z/A-L). Click here for file

Additional file 4 **Fig 8.4**. All cooperative articles that were produced between 1957 and 2007 are shown, (countries M- Z/M-Z). Click here for file
