# Supplementary material for: Scientometric analysis of the world-wide research efforts concerning Leishmaniasis
Source: Parasit Vectors. 2010 Mar 4;3:14. doi: 10.1186/1756-3305-3-14 (PMC2845575; doi:10.1186/1756-3305-3-14)
Supplement: Additional file 4 — Fig 8.4. All cooperative articles that were produced between 1957 and 2007 are shown, (countries M- Z/M-Z). [file 1756-3305-3-14-S4.PDF]

	Madagascar	Malawi	Malaysia	Mali	Malta	Martinique	Mexico	Monaco	Morocco	Namibia	Nepal	Netherlands	New Caledonia	New Zealand	Nicaragua	Niger	Nigeria	Norway	Oman	Pakistan	Panama	Papua New Guinea	Paraguay	Peru	Philippines	Poland	Portugal	Russia	San Marino	Saudi Arabia	Senegal	Singapore	Slovakia	Slovenia	Solomon Is.	Somalia	South Africa	South Korea	Spain	Sri Lanka	Sudan	Suriname	Sweden	Switzerland	Syria	Taiwan	Tanzania	Thailand	The Gambia	Togo	Trinidad & Tobago	Tunisia	Turkey	Turkmenistan	Uganda	Ukraine	United Arab Emirates	United Kingdom	United States	Uruguay	Uzbekistan	Venezuela	Vietnam	Yemen	Serbia	Zambia	Zimbabwe	
Madagascar																																																																				Madagascar
Malawi																													1											2																	1										Malawi
Malaysia																																											1														2										Malaysia
Mali															1						1																					5	3														1	4								1	Mali
Malta																																																									2										Malta
Martinique																																																																				Martinique
Mexico												3						1			1			1															4																			10	21			10						Mexico
Monaco																																																																				Monaco
Morocco																											1												4													1						2	1									Morocco
Namibia																																					1																															Namibia
Nepal																				2				3			2			1									1		1			12														6	4									Nepal
Netherlands							3													2				2		1	8	2									1		12		30	1	5	10			1	1				3	4					34	35			2	1					Netherlands
New Caledonia																							1																																													New Caledonia
New Zealand																																											1	4														2	2									New Zealand
Nicaragua																																							1				1															2	7			1						Nicaragua
Niger				1																																																						4	4									Niger
Nigeria																																																										4	4									Nigeria
Norway							1																																1				1	2														2	2									Norway
Oman																					1																																					3										Oman
Pakistan											2	2																													1	1		1									1					17	4				1					Pakistan
Panama							1												1																				2				4	1														4	18			1						Panama
Papua New Guinea				1																																								1														1									1	Papua New Guinea
Paraguay													1																										4									1										1	1	3		1						Paraguay
Peru							1				3	2																											6				1	10														27	19			1						Peru
Philippines																																																1																				Philippines
Poland												1																1											1				1	1														2	7									Poland
Portugal									1		2	8																											6		1			1									1					11	1									Portugal
Russia												2														1																	3											2				8	6		1							Russia
San Marino																																																										1										San Marino
Saudi Arabia		1									1																														4		2	3								1						12	4									Saudi Arabia
Senegal																																																										2	1									Senegal
Singapore																																											1					1										2	1			1						Singapore
Slovakia																																																											1									Slovakia
Slovenia																																																											1									Slovenia
Solomon Is.																																																																				Solomon Is.
Somalia																																											5																1									Somalia
South Africa										1		1																											1					3														18	11									South Africa
South Korea																																																											9									South Korea
Spain							4		4		1	12			1			1			2		4	6		1	6										1						4	17								1				1		46	42	4		17						Spain
Sri Lanka																																												1				1										4	3									Sri Lanka
Sudan		2									1	30								1							1			4													8	7								1					1	29	18		1			1				Sudan
Suriname												1								1																																	1						1									Suriname
Sweden				5								5		1	1			1			4			1		1		3		2		1				5			4		8			7														8	24	3	1	2						Sweden
Switzerland			1	3							12	10		4				2		1	1	1		10		1	1			3							3		17	1	7		7				3	1				3	8		1	1		79	91			5					2	Switzerland
Syria																																																										3										Syria
Taiwan																																																											8									Taiwan
Tanzania												1																																3														1										Tanzania
Thailand												1											1		1							1								1				1														5	3									Thailand
The Gambia																																																										2										The Gambia
Togo																																																																				Togo
Trinidad & Tobago																																																																				Trinidad & Tobago
Tunisia									1			3																		1									1		1			3														16	6									Tunisia
Turkey												4								1							1															1		8														2	9									Turkey
Turkmenistan																												2																														1										Turkmenistan
Uganda																																												1														1										Uganda
Ukraine																																							1					1																								Ukraine
United Arab Emirates																																									1																		2									United Arab Emirates
United Kingdom		1	2	1	2		10		2		6	34		2	2	4	4	2	3	17	4	1	1	27		2	11	8	1	12	2	2					18		46	4	29		8	79	3		1	5	2			16	2	1	1				215	2		37	2	3	2	1	1	United Kingdom
United States				4			21		1		4	35		2	7	4	4	2		4	18		1	19		7	1	6		4	1	1	1	1		1	11	9	42	3	18	1	24	91		8		3				6	9				2	215		4		47			1		1	United States
Uruguay																							3																4				3															2	4									Uruguay
Uzbekistan																												1													1		1																									Uzbekistan
Venezuela							10					2			1						1		1	1								1							17				2	5														37	47									Venezuela
Vietnam												1								1																																						2										Vietnam
Yemen																																									1																	3										Yemen
Serbia																																																										2	1									Serbia
Zambia																																																										1										Zambia
Zimbabwe				1																		1																						2														1	1									Zimbabwe

Madagascar Malawi Malaysia Mali Malta Martinique Mexico Monaco Morocco Namibia Nepal Netherlands New Caledonia New Zealand Nicaragua Niger Nigeria Norway Oman Pakistan Panama Papua New Guinea Paraguay Peru Philippines Poland Portugal Russia San Marino Saudi Arabia Senegal Singapore Slovakia Slovenia Solomon Is. Somalia South Africa South Korea Spain Sri Lanka Sudan Suriname Sweden Switzerland Syria Taiwan Tanzania Thailand The Gambia Togo Trinidad & Tobago Tunisia Turkey Turkmenistan Uganda Ukraine United Arab Emirates United Kingdom United States Uruguay Uzbekistan Venezuela Vietnam Yemen Serbia Zambia Zimbabwe
