# Supplementary material for: Scientometric analysis of the world-wide research efforts concerning Leishmaniasis
Source: Parasit Vectors. 2010 Mar 4;3:14. doi: 10.1186/1756-3305-3-14 (PMC2845575; doi:10.1186/1756-3305-3-14)
Supplement: Additional file 1 — Fig 8.1. All cooperative articles that were produced between 1957 and 2007 are shown, (countries A-L/A-M). [file 1756-3305-3-14-S1.PDF]

	Afghanistan	Albania	Algeria	Argentina	Australia	Austria	Azerbaijan	Bahrain	Bangladesh	Belgium	Belize	Benin	Bolivia	Brazil	Bulgaria	Burkina Faso	Cambodia	Cameroon	Canada	Chile	China	Colombia	Congo	Congo, DRC	Costa Rica	Cote dIvory	Croatia	Cuba	Cyprus	Czech Republic	Denmark	Djibouti	Dominica	Dominican Republic	Ecuador	Egypt	El Salvador	Estonia	Ethiopia	Finland	France	French Guiana	Gabon	Georgia	Germany	Ghana	Greece	Guatemala	Guinea	Guinea-Bissau	Guyana	Honduras	Hungary	India	Indonesia	Iran	Iraq	Ireland	Israel	Italy	Japan	Jordan	Kenya	Lebanon	Libya	Lithuania	Luxembourg	
Afghanistan																																													1												1												Afghanistan
Albania																																																												2								Albania
Algeria									1																															10																								1				Algeria
Argentina				1					2			2	23					1	7		1						2			2										13				6														1	1	7							Argentina
Australia			1									1	2					8		3							1			7										6		1		30									3	1	1		1	4		3		4					Australia
Austria										3									1											1					1				1		3				14			2						2					1									Austria
Azerbaijan																																																																				Azerbaijan
Bahrain																																				1																																	Bahrain
Bangladesh																																																													5							Bangladesh
Belgium			1	2		3						1	9	12					6	1							1			4									2	3	41				22		4							5				1	1	4			4	2				Belgium
Belize																																																											1									Belize
Benin										1								1																							1																											Benin
Bolivia				2	1					9				4								5													1					39	1			2																							Bolivia
Brazil				23	2					12			4						1	22	1		16						5		2	6				2		1				71	1			22		1	1						6	1				4	8	7		3					Brazil
Bulgaria																																															1																					Bulgaria
Burkina Faso																																									2																											Burkina Faso
Cambodia																																																																				Cambodia
Cameroon													1		1																												5				1																						Cameroon
Canada				1	8	1				6				22							2	6								1	4								6		17				21								6		12			5	2	8		6					Canada
Chile				7						1				1								1																				13																										Chile
China					3														2		1										1										1				1								1							4							China
Colombia				1										5	16					6	1	1							1														10				11			1						1					1	1									Colombia
Congo																												1																					3																							Congo
Congo, DRC																												1																					2																							Congo, DRC
Costa Rica																															1										1							1				3																	Costa Rica
Cote dIvory																									1	1																			1				1																						Cote dIvory
Croatia										1																															2				1															1									Croatia
Cuba				2	1										5									1																																																1			Cuba
Cyprus																																									2																			2								Cyprus
Czech Republic						1				4				2					1							1					1						1					7				8		2												4	2	1							Czech Republic
Denmark				2	7											6					4			1																					1	1				7	1											9				2	1		14					Denmark
Djibouti																																									1																											Djibouti
Dominica																																																																				Dominica
Dominican Republic																																																																				Dominican Republic
Ecuador						1								1	2																												6				1																26							Ecuador
Egypt								1																									1														4					2									1				2	2									Egypt
El Salvador																1																																																					El Salvador
Estonia																																													1																							Estonia
Ethiopia						1				2										6																							3				2		1							2						2			2					Ethiopia
Finland										3																					1																2																							Finland
France			10	13	6	3				41		1	39	71		2		5	17	13	1	10			1	1	2		2	7	1	1			6	4			3			21	4	2	41		22							8		2			11	14	6	1	3	2			1	France
French Guiana													1	1																													21				1											1					2							French Guiana
Gabon					1																																					4				3																							Gabon
Georgia																																									2																											Georgia
Germany	1			6	30	14				22			2	22				1	21		1	11	3	2		1	1			8	7				1			1	2	2	41	1	3			1	4	3					2	28		2			30	14							1	1	Germany
Ghana																															1					2									1																				1					Ghana
Greece										4				1	1																2									1		22				4										1				1	2	3									Greece
Guatemala						2									1								1			1																									3								2																		Guatemala
Guinea																																																												1								Guinea
Guinea-Bissau																																																												1								Guinea-Bissau
Guyana																																																																				Guyana
Honduras																									3																									2																					Honduras
Hungary																																													2									1																Hungary
India					3	2				5				6					6		1	1																	2		8				28		1						1						4	2	1		4					India
Indonesia					1									1																						1																																	Indonesia
Iran	1				1														12											9										2	1			2															1	2									Iran
Iraq																																																																				Iraq
Ireland					1					1																																					1																					Ireland
Israel				1	4					1	1			4					5			1								4							2					11				30		2							4		1				3	1		4					Israel
Italy		2		1		1				4				8					2			1					1		2	2	2						2			2		14				14		3		1	1				2		2			3		1	2	2				1	Italy
Japan				7	3				5						7					8			4										1	1				26						6	2			18											1				1	1			4					Japan
Jordan																																									1																				2								Jordan
Kenya					4					4				3					6												14								2		3				5	1								4				4	2	4							Kenya
Lebanon			1							2																															2																											Lebanon
Libya																												1																																									Libya
Lithuania																																													1																							Lithuania
Luxembourg																																									1				1																1								Luxembourg
	Afghanistan	Albania	Algeria	Argentina	Australia	Austria	Azerbaijan	Bahrain	Bangladesh	Belgium	Belize	Benin	Bolivia	Brazil	Bulgaria	Burkina Faso	Cambodia	Cameroon	Canada	Chile	China	Colombia	Congo	Congo, DRC	Costa Rica	Cote dIvory	Croatia	Cuba	Cyprus	Czech Republic	Denmark	Djibouti	Dominica	Dominican Republic	Ecuador	Egypt	El Salvador	Estonia	Ethiopia	Finland	France	French Guiana	Gabon	Georgia	Germany	Ghana	Greece	Guatemala	Guinea	Guinea-Bissau	Guyana	Honduras	Hungary	India	Indonesia	Iran	Iraq	Ireland	Israel	Italy	Japan	Jordan	Kenya	Lebanon	Libya	Lithuania	Luxembourg	
