# Supplementary material for: Scientometric analysis of the world-wide research efforts concerning Leishmaniasis
Source: Parasit Vectors. 2010 Mar 4;3:14. doi: 10.1186/1756-3305-3-14 (PMC2845575; doi:10.1186/1756-3305-3-14)
Supplement: Additional file 2 — Fig 8.2. All cooperative articles that were produced between 1957 and 2007 are shown, (countries A-L/M-Z). [file 1756-3305-3-14-S2.PDF]

	Afghanistan	Albania	Algeria	Argentina	Australia	Austria	Azerbaijan	Bahrain	Bangladesh	Belgium	Belize	Benin	Bolivia	Brazil	Bulgaria	Burkina Faso	Cambodia	Cameroon	Canada	Chile	China	Colombia
Madagascar													1									
Malawi																						
Malaysia																						
Mali			1											1								
Malta																						
Martinique																						
Mexico				2	1					5			3	5								1
Monaco																						
Morocco																						
Namibia																						
Nepal					1					18								1				
Netherlands					2	1			3	16				10					9	1		
New Caledonia																						
New Zealand					1																1	
Nicaragua																						
Niger				1										1				2			1	
Nigeria				1										1				2			1	
Norway																			2			
Oman																						
Pakistan	1			1						1				2			1	2				
Panama				1										4						1		
Papua New Guinea																						
Paraguay				3									3							6		
Peru										22			8	5					8			1
Philippines																						
Poland					1														1			
Portugal										6				4								
Russia							1							2					2			
San Marino																						
Saudi Arabia														1					1			
Senegal																						
Singapore				1										1								
Slovakia																						
Slovenia				3																		
Solomon Is.									1													
Somalia																						
South Africa										5									1			
South Korea												1		1				1			1	
Spain				12	2	4				14			3	19				2	5	5		27
Sri Lanka														1								
Sudan					1				1	4				6					1			
Suriname														1								
Sweden				13	1					2			1	10					8	9	1	
Switzerland			1	1	9	2				31		1	7	21				3	7		1	8
Syria						1								1					1			
Taiwan																						
Tanzania										2												
Thailand										1				3								
The Gambia																						
Togo																						
Trinidad & Tobago																						
Tunisia			2							8				1								
Turkey														2								
Turkmenistan																						
Uganda										1												
Ukraine																						
United Arab Emirates														1								
United Kingdom	4			10	35	7				41	1		3	145			1	1	26		6	19
United States	2			19	38	6		1	9	28			5	364				2	148	2	10	63
Uruguay				2	1									2						3		
Uzbekistan																			1			
Venezuela				7						7			3	37					5			9
Vietnam										4							1					
Yemen																						
Serbia					1																	
Zambia																						
Zimbabwe																						

	Congo	Congo, DRC	Costa Rica	Cote dIvory	Croatia	Cuba	Cyprus	Czech Republic	Denmark	Djibouti	Dominica	Dominican Republic	Ecuador	Egypt	El Salvador	Estonia	Ethiopia	Finland	France	French Guiana	Gabon	Georgia	Germany
Madagascar																			1				
Malawi																							1
Malaysia																							
Mali								1									1		3				
Malta																							
Martinique																			1				
Mexico						1			1								1		4				6
Monaco																			2				
Morocco																			15				
Namibia																							
Nepal																			2				3
Netherlands								7	3					1			12	1	13		1		22
New Caledonia																			2				
New Zealand																							6
Nicaragua			2										1				1		1				
Niger																			2				
Nigeria																			1				
Norway																	4		3				
Oman																							
Pakistan																			1				2
Panama											1	1		1									
Papua New Guinea																							
Paraguay																			14				3
Peru									2				1				1		16				1
Philippines																							
Poland								1															1
Portugal								2	1					1					27				8
Russia			1					2											4				7
San Marino																							
Saudi Arabia														5					2				
Senegal																			4	1			
Singapore																							
Slovakia								2											1				
Slovenia					1																		1
Solomon Is.																							
Somalia																	1						
South Africa																			3				12
South Korea																			3				
Spain			1		1	3		1					1	1			1	2	47	1			11
Sri Lanka																			1				
Sudan									34								2		13				5
Suriname																							
Sweden									2								22		12				7
Switzerland	4	2		1			1	2	3				1	1			8	2	52	5			54
Syria																			2				5
Taiwan																							
Tanzania									2														2
Thailand																			1				1
The Gambia																							
Togo																							2
Trinidad & Tobago																							
Tunisia														1					22				
Turkey								4															3
Turkmenistan																							
Uganda																			1				
Ukraine																							
United Arab Emirates																							
United Kingdom	1		2			1		6	15				3	2			11	5	92	1			91
United States			7		1			30	4		4	4	12	10	2		5		103	2	3		134
Uruguay																			1				3
Uzbekistan																			2				
Venezuela			1																11				12
Vietnam																							
Yemen																							
Serbia																			1				
Zambia																							
Zimbabwe																							

	Ghana	Greece	Guatemala	Guinea	Guinea-Bissau	Guyana	Honduras	Hungary	India	Indonesia	Iran	Iraq	Ireland	Israel	Italy	Japan	Jordan	Kenya	Lebanon	Libya	Lithuania	Luxembourg
Madagascar																						
Malawi																						
Malaysia									1	1						2						
Mali				1												1						
Malta																						
Martinique																						
Mexico														2	1							
Monaco																						
Morocco		1												3								
Namibia																						
Nepal									2						1	1		1				
Netherlands		1					1		5				1	6	7		2	16				
New Caledonia																						
New Zealand														1	1	3						
Nicaragua			2				2								1							
Niger									1													
Nigeria									1													
Norway									1				1									
Oman																						
Pakistan																3						
Panama															1	1						
Papua New Guinea																						
Paraguay																3						
Peru																						
Philippines																						
Poland		1												3	1							
Portugal									1					3	3							
Russia									1					3								
San Marino															1							
Saudi Arabia															3							
Senegal																						
Singapore																						
Slovakia																						
Slovenia		1																				
Solomon Is.																1						
Somalia																						
South Africa														1				3				
South Korea																1						
Spain		2	2					1	4	1	3			6	12	3						
Sri Lanka									1									1				
Sudan									1									2				
Suriname																						
Sweden							1		4		2			2	3	2						
Switzerland	1	2		1			1		18	1	12			5	12	4		7	1			1
Syria														2	1							
Taiwan																1						
Tanzania																						
Thailand									1							1		1				
The Gambia																						
Togo																						
Trinidad & Tobago						1																
Tunisia		1													3			1	2	1		
Turkey														2	1							
Turkmenistan																						
Uganda																		1				
Ukraine																						
United Arab Emirates																						
United Kingdom		8	5	1		1	4	3	43		17		3	21	38	11	1	17	2	2		
United States	4	14	7				5	2	103	5	10	2	3	44	26	58	2	37		2	1	
Uruguay																						
Uzbekistan													1		1							
Venezuela			1						2					5	5							
Vietnam																						
Yemen															1							
Serbia		2																				
Zambia																						
Zimbabwe				1																		
